# Core functionalization of semi-crystalline polymeric cylindrical nanoparticles using photo-initiated thiol–ene radical reactions†
†Electronic supplementary information (ESI) available: Further methods, polymer and nanostructure characterization. See DOI: 10.1039/c5py01970b



**DOI:** 10.1039/c5py01970b

**Published:** 2016-02-25

**Authors:** Liang Sun, Anaïs Pitto-Barry, Anthony W. Thomas, Maria Inam, Kay Doncom, Andrew P. Dove, Rachel K. O'Reilly

**Affiliations:** a Department of Chemistry , University of Warwick , Gibbet Hill Road , Coventry , CV4 7AL , UK . Email: a.p.dove@warwick.ac.uk ; Email: r.k.o-reilly@warwick.ac.uk; b Department of Materials Engineering , Monash University , Clayton , Victoria 3800 , Australia

## Abstract

Sequential ring-opening and reversible addition–fragmentation chain transfer (RAFT) polymerization was used to form a triblock copolymer of tetrahydropyran acrylate (THPA), 5-methyl-5-allyloxycarbonyl-1,3-dioxan-2-one (MAC) and l-lactide. Concurrent deprotection of the THPA block and crystallization-driven self-assembly (CDSA) was undertaken and allowed for the formation of cylindrical micelles bearing allyl handles in a short outer core segment. These handles were further functionalized by different thiols using photo-initiated thiol–ene radical reactions to demonstrate that the incorporation of an amorphous PMAC block within the core does not disrupt CDSA and can be used to load the cylindrical nanoparticles with cargo.

## Abstract 2

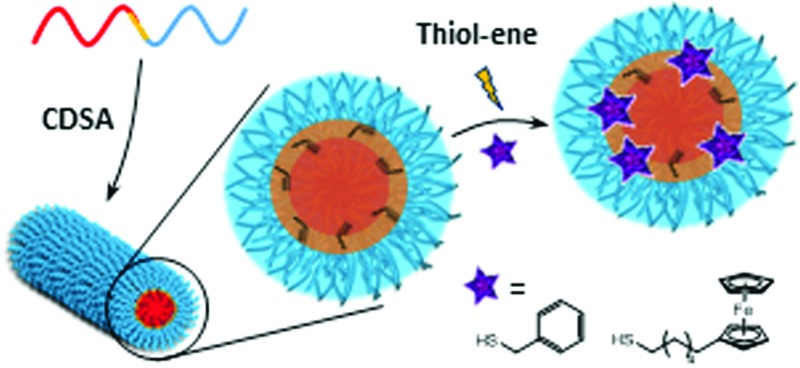
Functionalisation by radical thiol–ene addition of a cylindrical micelle formed by crystallisation-driven self-assembly is demonstrated.

To afford nanoparticles with different features and functions, chemical modification is often required. Functionalized nanoparticles can be achieved either by modification of the precursor amphiphilic polymers followed by self-assembly or by direct functionalization of the self-assembled nanostructures.^1–7^ The former approach, although it is more flexible to design a polymer for a specific target, can be time-consuming and often requires multiple synthetic steps. In contrast, the post-assembly functionalization on micellar scaffolds is a faster and more versatile route as it avoids the synthesis and polymerization of the new functional monomers and optimization of multiple self-assembly conditions. Click-type reactions such as the Diels–Alder reaction,^8^ thiol–ene reaction,^9,10^ copper-catalyzed azide–alkyne cycloaddition (CuAAC) reaction^11^ and tetrazine–norbornene reaction^12^ are often used for the chemical modifications. Among them, the thiol–ene radical reaction displays outstanding reaction properties as it has a rapid reaction rate and allows reactions between a wide range of thiols and alkenes and it is largely insensitive to oxygen or water.^13^ Thiol–ene radical reactions have been applied to numerous areas including the functionalization of polymers,^14–16^ modification of substrate surfaces^17^ and preparation of hydrogels.^18^

Polylactide (PLA) is a well-known biodegradable and biocompatible polymer that has been widely studied as a biomaterial.^19–23^ We have previously demonstrated that the semi-crystalline nature of PLA enables its use to direct crystallization-driven self-assembly (CDSA) to readily access cylindrical nanoparticles.^24–29^ However, the lack of functional handles makes the encapsulation of small molecules into this crystalline environment challenging and potentially limits the application of these nanoparticles in delivery applications. The ROP of functional glycolide monomer has been reported, however monomer synthesis often requires tedious synthetic procedures.^30–32^ An alternative approach to introduce functional handles into PLAs is to copolymerize lactide with functionalized carbonate monomers using ROP methods.^9,33–36^ Cyclic carbonate monomers have been widely reported and are readily synthesized through a variety of routes.^37–39^

In order to overcome the limitations of the crystalline core for physical encapsulation and the lack of ready incorporation of functional handles into semi-crystalline block copolymers^29,40^ that may undergo CDSA, we postulated that the introduction of a third, amorphous, block in the copolymer will enable introduction of the desired functional group alongside CDSA. Few examples have been reported on the CDSA of triblock copolymers. In most cases the semi-crystalline or crystalline block is the middle block as in the ABC or ABA copolymers.^41,42^ Varying the A and C blocks allows the tuning of microphase separation and therefore the surface compartmentalization.^43,44^ Miktoarm star terpolymers made with a core-forming polyferrocenylsilane block exhibited a transition from spherical to rod-like micelles upon ageing *via* a CDSA process.^45^ Triblock copolymers have also been used by Manners, Winnik and coworkers in combination with diblock copolymers to obtain more complex cylindrical micelles.^46,47^ In both examples the second corona-forming block was used to functionalize the shell of the particles for imaging.

Herein, we report the synthesis of a triblock copolymer that allows the encapsulation of small hydrophobic molecules inside the assembled cylindrical nanostructure through conjugation to the polymer backbone. To this end, the subsequent polymerization of l-lactide (l-LA) and an allyl functional cyclic carbonate, 5-methyl-5-allyloxycarbonyl-1,3-dioxan-2-one (MAC) by ring-opening polymerization (ROP), followed by reversible addition–fragmentation chain-transfer (RAFT) polymerization of tetrahydropyran acrylate (THPA) from a dual-headed initiator yields PLLA-*b*-PMAC-*b*-PTHPA triblock copolymer that contains four allyl groups per unimer chain that are suitable for post-assembly modification. Following self-assembly into cylindrical nanoparticles by CDSA we demonstrate that the core can be functionalized with thiols, benzyl mercaptan and 6-(ferrocenyl)hexanethiol using photo-initiated thiol–ene radical reactions.

The PLLA-*b*-PMAC-*b*-PTHPA triblock copolymer was synthesized in a manner similar to our previous reports of PLLA-*b*-PTHPA diblock copolymers^24,25,27,28^ (Scheme 1). Firstly, ROP of MAC was carried out using an organic co-catalyst system comprised of 1-(3,5-bis(trifluoromethyl)phenyl)-3-cyclohexyl-thiourea, **1**, and (–)-sparteine^48^ from the dual-headed initiator, **2**. The targeted degree of polymerization (DP) of PMAC was 4 and after just 1 h the conversion of the MAC monomer had reached 92%, as confirmed by ^1^H NMR spectroscopy (Fig. S1†). A solution of l-LA in CDCl_3_ was then added to the crude PMAC solution with a further addition of **1** and (–)-sparteine. After 3 h, the conversion of the l-LA monomer reached 90% as confirmed by ^1^H NMR spectroscopy and the polymerization was stopped. ^1^H NMR spectroscopic analysis confirmed the successful synthesis of PMAC-*b*-PLLA diblock copolymer, **3**, with the methylene resonance of the PMAC backbone observed at *δ* = 4.45–4.22 ppm and the methine resonance of the PLLA repeat units observed at *δ* = 5.36–4.96 ppm (Fig. S2†). SEC analysis showed a narrow dispersity for the PLLA-*b*-PMAC diblock copolymer (*Đ*_M_ = 1.08). PLLA-*b*-PMAC diblock copolymer, **3**, possessed a *T*_g_ at 45 °C and a *T*_m_ at 141 °C as measured by differential scanning calorimetry (DSC) analysis (Fig. S3†), which correspond to those expected for PLLA. The absence of a second *T*_g_ at *ca.* –27 °C from the PMAC block is most likely a result of the low DP of the PMAC block and is not necessarily an indication of an absence of phase separation.

**Scheme 1:**
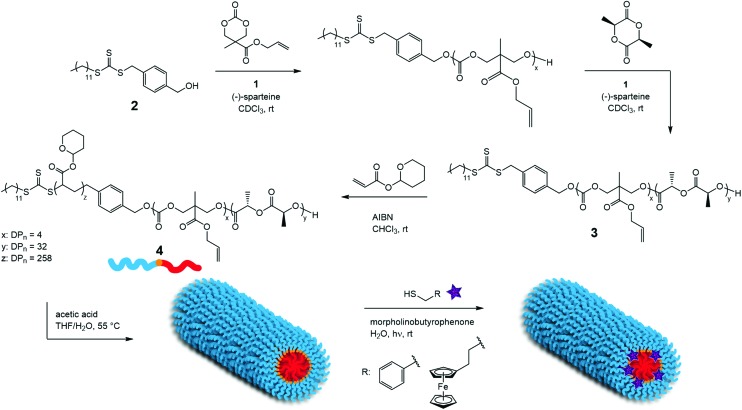
Synthetic procedures of PLLA-*b*-PMAC-*b*-PTHPA triblock copolymer, **2**, its crystallization-driven self-assembly and the further internal functionalization of the PMAC block *via* thiol–ene radical reaction.

THPA was then polymerized from the PLLA-*b*-PMAC macro-initiator, **3**, using RAFT polymerization (Scheme 1). ^1^H NMR spectroscopic analysis indicated the successful synthesis of PLLA_32_-*b*-PMAC_4_-*b*-PTHPA_258_ triblock copolymer, **4**, with the broad methine resonance of the tetrahydropyranyl protecting groups of the PTHPA repeat units at *δ* = 6.20–5.70 ppm (Fig. 1). Based on our previous results,^25^ the triblock copolymer was designed with a DP of PLLA of 32 and a hydrophobic weight fraction of 22.5% in order to access well-defined cylindrical nanostructures. SEC analysis revealed a dispersity for the triblock copolymer of 1.27 and the successful chain-extension from PLLA-*b*-PMAC macro-initiator, **3** (Fig. 2 and Table 1).

**Fig. 1:**
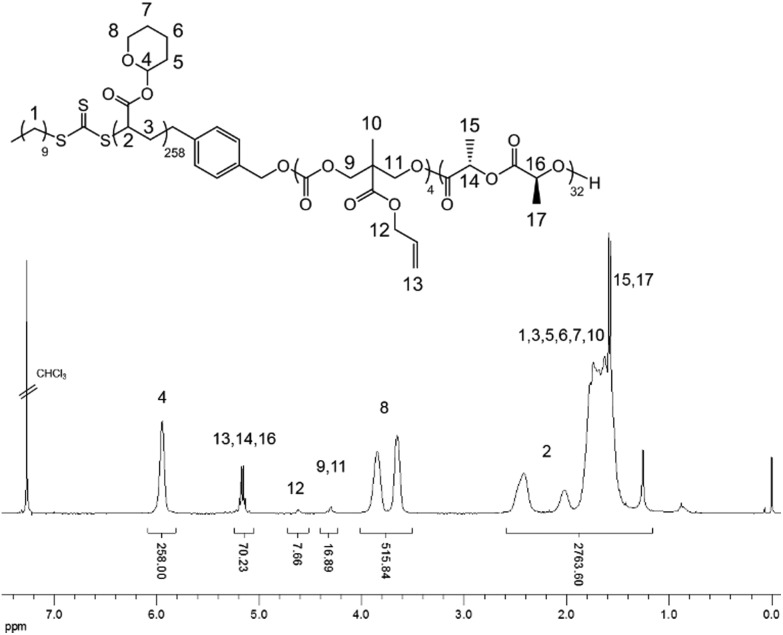
^1^H NMR spectrum (400 MHz, CDCl_3_) of PLLA-*b*-PMAC-*b*-PTHPA triblock copolymer, **4**.

**Fig. 2:**
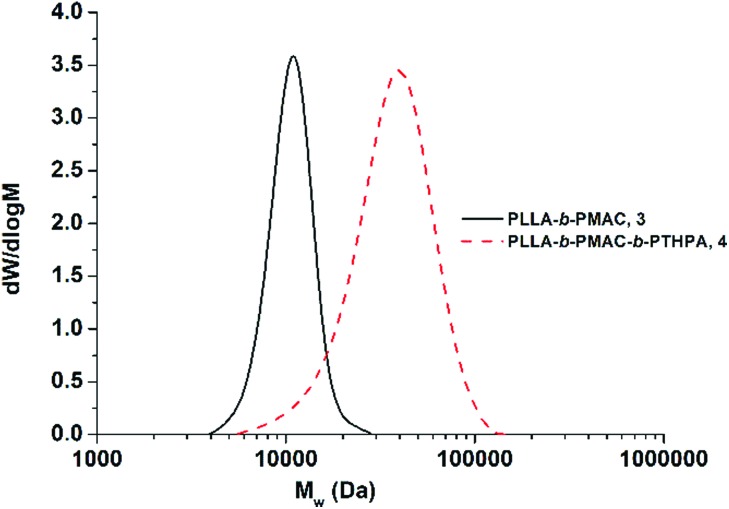
SEC chromatograms (CHCl_3_ with 0.5% TEA as eluent, RI detection) of PLLA-*b*-PMAC diblock copolymer, **3** and PLLA-*b*-PMAC-*b*-PTHPA triblock copolymer, **4**.

**Table 1:** Characterization of PLLA-*b*-PMAC, **3** and PLLA-*b*-PMAC-*b*-PTHPA, **4**

Polymer	*M*_n_ ^*a*^ (kDa)	*Đ*_M_ ^*b*^	*T*_g_ ^*c*^ (°C)	Hydrophobic wt%^*d*^
PLLA_32_-*b*-PMAC_4_, **3**	5.8	1.08	45	100
PLLA_32_-*b*-PMAC_4_-*b*-PTHPA_258_, **4**	46.1	1.27	—	22.5

^*a*^Measured by ^1^H NMR spectroscopy (400 MHz, CDCl_3_).

^*b*^Measured by SEC analysis (CHCl_3_ with 0.5% TEA as eluent).

^*c*^Measured by DSC analysis, with a heating and cooling rate of 10 °C min^–1^.

^*d*^PLLA-*b*-PMAC weight fraction in the PLLA-*b*-PMAC-*b*-PAA triblock copolymer.

The CDSA of the PLLA-*b*-PMAC-*b*-PTHPA triblock copolymer, **4**, was performed in a mixture of THF/H_2_O (v : v = 20 : 80) at 55 °C, which is above the *T*_g_ of the core block. During the time scale of the experiment, the evaporation of THF occurs and after 30 h, well-defined PLLA-*b*-PMAC-*b*-PAA cylindrical micelles were obtained. Analysis by dynamic light scattering (DLS) displayed only one size population of the assemblies (Fig. S4†) and analysis by transmission electron microscopy (TEM) confirmed the cylindrical structure that was expected (*L*_n_ = 221 nm, *L*_w_/*L*_n_ = 1.17, *W*_n_ = 52 ± 5 nm, Fig. 3, *L*: length and *W*: width of cylindrical micelles). An intense crystalline Bragg peak at a 2*θ* value of 16.6° was observed in the WAXD diffractogram (Fig. S5†), which confirms the crystalline nature of the core of these nanoparticles.

**Fig. 3:**
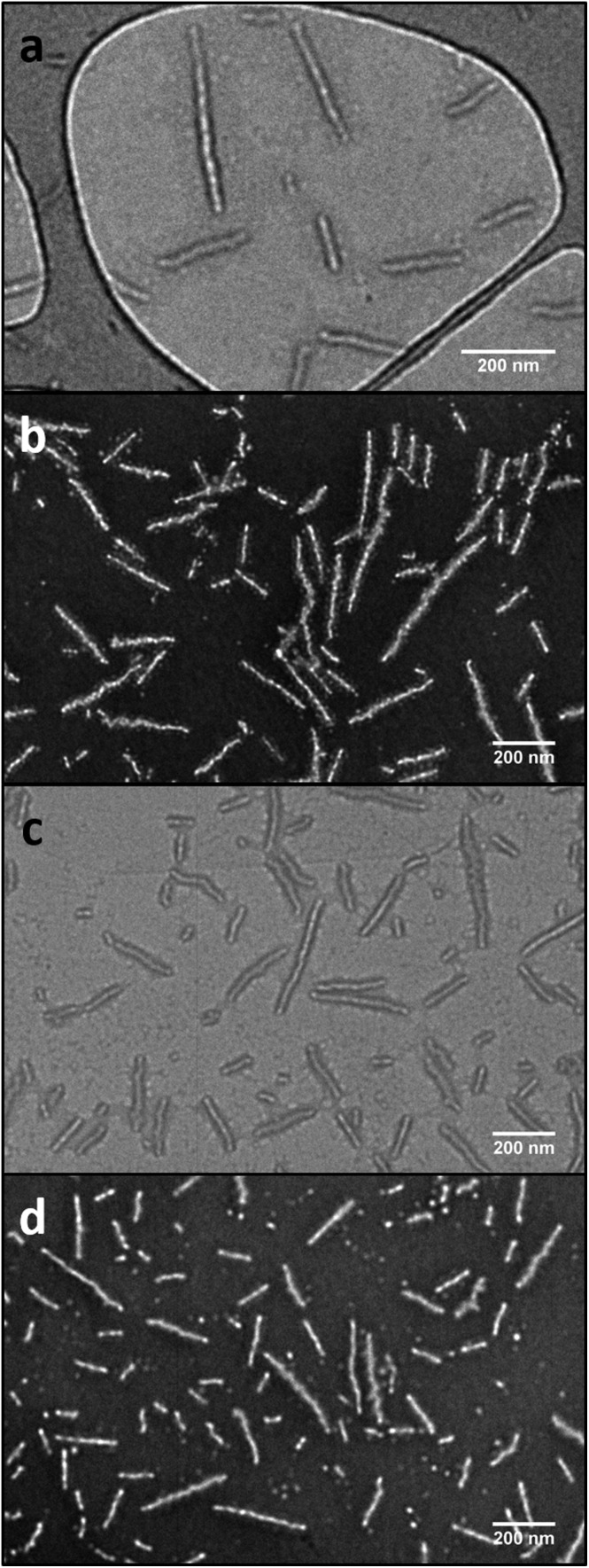
TEM images showing the PLLA-*b*-PMAC-*b*-PAA cylindrical micelles before (a and b) and after functionalization with benzyl mercaptan (c and d). a and c, on graphene oxide grid;^49^ b and d, with negative staining using phosphotungstic acid (PTA). Scale bar = 200 nm.

In order to confirm that the PMAC within the core of the cylindrical micelles can still be functionalized, a photo-initiated radical thiol–ene addition^16^ of benzyl mercaptan under a range of different conditions was performed (Table S1†). Using a mixture of an aqueous solution of cylindrical micelles, benzyl mercaptan (10 eq. to each allyl group) and 2-benzyl-2-(dimethylamino)-4′-morpholinobutyrophenone as a UV photo-initiator, exposure to UV irradiation for 1 h followed by exhaustive dialysis against an aqueous solution (resistivity 18.2 MΩ cm) containing 2% of 1,4-dioxane before subsequent lyophilization enabled isolation of core-functionalized nanoparticles. In order to characterize the functionalized nanoparticles, ^1^H NMR spectroscopic analysis was undertaken of the freeze-dried nanoparticles dissolved in *d*_6_-DMSO (Fig. 4, Table S1 and Fig. S6†). A functionalization ratio of 74% was calculated from the ratio of characteristic aromatic resonance of benzyl mercaptan observed at *δ* = 7.46–7.14 ppm to polymer backbone resonances at *δ* = 4.70–4.00 ppm. To allow benzyl mercaptan and the UV initiator an easier access to the core of the cylindrical micelles to target a higher functionalization ratio, a small amount of THF or 1,4-dioxane (5% v/v) was added into the reaction mixture and the functionalization repeated (Table S1†) however no improvement in functionalization degree was observed. It is proposed that some allyl groups can be physically trapped in the crystalline PLLA core of the cylindrical micelles during CDSA which would make complete functionalization of the allyl groups not possible. Notably, in comparison, the unassembled PLLA-*b*-PMAC-*b*-PAA triblock copolymer can be easily modified with benzyl mercaptan to obtain a full functionalization ratio using the photo-initiated thiol–ene radical reaction under comparable conditions (Fig. S7†) which further proved our hypothesis that the allyl groups of PMAC in the cylindrical nanoparticles were physically inaccessible. TEM analysis confirmed that the PLLA-*b*-PMAC-*b*-PAA cylindrical micelles did retain their size and morphology after functionalization with benzyl mercaptan (Fig. 3c and d) and the dimensions remained nearly the same (*L*_n_ = 217 nm, *L*_w_/*L*_n_ = 1.12, *W*_n_ = 55 ± 7 nm). The crystallinity of the cylindrical micelles is not disturbed as confirmed by WAXD (Fig. S5†).

**Fig. 4:**
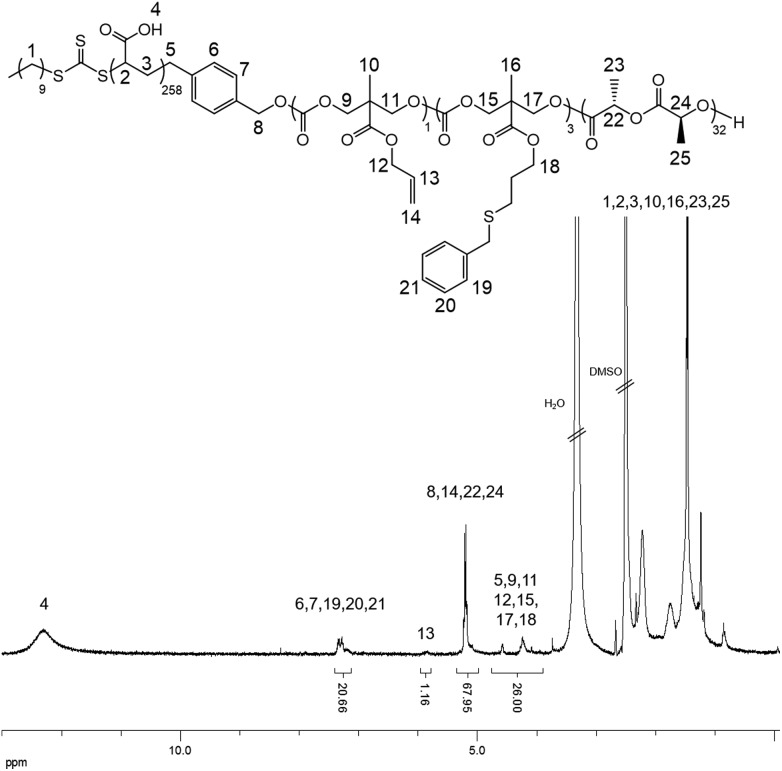
^1^H NMR spectrum (500 MHz, *d*_6_-DMSO) of benzyl mercaptan functionalized PLLA-*b*-PMAC-*b*-PAA cylindrical micelles.

To further prove the accessibility of the allyl groups inside the micelles, a more bulky thiol molecule, 6-(ferrocenyl)hexanethiol, was used for the photo-initiated thiol–ene radical reaction. A lower functionalization ratio of 49% was obtained when compared to that of benzyl mercaptan (Table S1 and Fig. S8†). It is more likely that 6-(ferrocenyl)hexanethiol was not dispersed enough in the aqueous solution of cylinders to access the core domain since this thiol is a viscous oil. TEM images of the ferrocenyl-functionalized cylinders also confirmed the non-destruction of the morphology after functionalization (Fig. S9†).

## Conclusions

In summary, we have successfully prepared PLLA-*b*-PMAC-*b*-PTHPA triblock copolymers using a combination of ROP and RAFT polymerization. Well-defined cylindrical micelles were obtained from the CDSA of the triblock copolymers. By using photo-initiated thiol–ene radical reactions, benzyl mercaptan and 6-(ferrocenyl)hexanethiol were “clicked” onto the allyl groups of PMAC in the self-assembled PLLA-*b*-PMAC-*b*-PAA cylindrical micelles, showing that the core functionalization of these triblock cylinders is possible. The embedment of a functional segment, the short PMAC block, was used to encapsulate some hydrophobic small molecules within the hydrophobic core of the cylindrical micelles without disturbing their self-assembly. This opens up new pathways for the delivery of hydrophobic drugs capable of release by degradation of the core block *via* robust micellar carrier nanoparticles that are not subject to disintegration upon dissolution.
