# Supplementary material for: Core functionalization of semi-crystalline polymeric cylindrical nanoparticles using photo-initiated thiol–ene radical reactions
Source: Polym Chem. 2016 Feb 25;7(13):2337–41. doi: 10.1039/c5py01970b (PMC4936379; doi:10.1039/c5py01970b)
Supplement: Supplementary file 1 [file PY-007-C5PY01970B-s001.pdf]

Electronic Supplementary Material (ESI) for Polymer Chemistry.
This journal is © The Royal Society of Chemistry 2016

Supplementary Information

for

Core Functionalization of Semi-Crystalline Polymeric Cylindrical Nanoparticles Using Photo-initiated Thiol-ene Radical Reactions

Liang Sun,[a] Anaïs Pitto-Barry,[a] Anthony W. Thomas,[a] Maria Inam,[a] Kay Doncom,[a] Andrew P. Dove*[a,b] and Rachel K. O'Reilly*[a]

Experimental section

Materials

Chemicals and solvents were used as purchased from Aldrich, Acros, Fluka, Fisher Chemical, Alfa Aesar or VWR. *L*-Lactide (*L*-LA) monomer was kindly donated by Corbion-Purac and was passed through a silica plug with dichloromethane as eluent to remove impurities and then dried over 3 Å molecular sieves in CH_2Cl_2. *L*-LA monomer was further purified by recrystallization in toluene before being stored in a glove box under an inert atmosphere. (-)-Sparteine was dried over CaH_2 before use. 1-(3,5-Bis(trifluoromethyl)phenyl)-3-cyclohexyl-thiourea, **1**,[1] and THPA were synthesized and purified as described previously.[2, 3] 5-Methyl-5-allyloxycarbonyl-1,3-dioxan-2-one (MAC) was synthesized as reported[4] and dried over 3 Å molecular sieves in CH_2Cl_2. 2,2-Azobis(isobutyronitrile) (AIBN) was recrystallized from methanol and stored at 4 °C. The chain transfer agent dodecyl 4-(hydroxymethyl) benzyl carbonotrithioate, **2**, was synthesized and purified as previously described.[5]

Instrumentations

^1H Nuclear magnetic resonance (^1H NMR) spectra were recorded on a Bruker spectrometer operating at a frequency of 400 MHz in $CDCl_3$ (unless stated otherwise). The chemical shifts are given in ppm with tetramethylsilane (TMS) as an internal reference.

Size exclusion chromatography (SEC) was performed on an Agilent 1260 Infinity Multi-Detector SEC instrument equipped with refractive index and UV detectors with $CHCl_3$ and 0.5% triethylamine as eluent at a flow rate of 1 mL/min. SEC data was calibrated by Cirrus GPC software with PS standards.

Differential scanning calorimetry (DSC) analysis was performed using a Mettler Toledo DSC1 star system. Samples were run at a heating or cooling ramp of 10 °C·min^{-1} in triplicate in series under a nitrogen atmosphere in 40 µL aluminum crucibles. T_g and T_m of various samples were obtained in the first runs.

The stained transmission electron microscopy (TEM) images were obtained by using a JEOL 2000FX instrument operated at 200 kV. TEM samples were negatively stained by phosphotungstic acid (PTA,

2 wt%) or uranyl acetate (UA, 2.5 wt%) on formvar/carbon grids (300 Mesh, Cu, Elektron Technology UK LTD). Typically, formvar/carbon grids were cleaned by air plasma from a glow-discharge system (2 min, 20 mA) which also improved the hydrophilicity of the grids. 20 µL of particle solution (0.25 mg/mL) was added onto the grid and the solution was blotted away after 2 min and then left to air-dry. 5 µL of a 2 wt% PTA solution was then added onto the grid to stain the particles and was blotted away after 30 s before air-drying.

TEM images on graphene oxide (GO) support were also obtained using a JEOL 2000FX instrument operated at 200 kV. The GO grids were prepared as follows: lacey carbon grids (400 Mesh, Cu, Elektron Technology UK LTD) were cleaned by air plasma from a glow-discharge system (2 min, 20 mA) to improve the hydrophilicity of the lacey carbon. One drop of GO solution (0.10 - 0.15 mg·mL^{-1}) was deposited on each grid and left to air-dry totally. Then one drop of the sample solution (20 µL) was added onto a GO grid and after 2 min, the solution was blotted away before drying totally.

The hydrodynamic diameter (D_h) of nanoparticles was determined by dynamic light scattering (DLS). Typically, scattering of a 0.25 mg·mL^{-1} aqueous nanoparticle solution was measured with a Malvern Zetasizer NanoS instrument equipped with a 4 mW He-Ne 633 nm laser module at 25 °C. Measurements were carried out at a detection angle of 173° (back scattering) and the data was further analyzed by Malvern DTS 6.20 software. D_h was calculated by fitting the apparent diffusion coefficient in the Stokes-Einstein equation $D_h = kT/(3\pi\eta D_{app})$, where k is the Boltzmann constant, T is the temperature and η is the viscosity of the solvent. D_h only coincides to the real hydrodynamic diameter when the measured sample is a solution of monodispersed spherical particles as D_{app} equals the translational diffusion (D_t). For cylindrical particles, owing to their anisotropy, the rotational diffusion is not negligible and contributes to the D_{app}. Therefore, the D_h measured for the cylindrical micelles only has a relative value and provides dispersity information to detect multiple populations.

WAXD was performed on a Panalytical X'Pert Pro MPD equipped with a CuKα_1 hybrid monochromator as the incident beam optics. Generally, *ca*. 30 mg of self-assembled freeze-dried particles were placed in a 10 mm sample holder, and standard "powder" 2ϑ - ϑ diffraction scans were carried out in the angular range from 2ϑ 10° to 30° at room temperature.

Photo-initiated thiol-ene radical reactions were carried out in a Metalight QX1 light box equipped with 12 × 9 W bulbs with a peak output at 365 nm for 1 h. Typically, samples were placed 10 cm away from the source with the bulbs arranged concentrically around them.

Synthesis of PMAC homopolymer and PLLA-*b*-PMAC diblock copolymer, 3.

$PMAC_4$ was synthesized in a glove box under nitrogen atmosphere using ROP from the dual-headed initiator, dodecyl 4-(hydroxymethyl) benzyl carbonotrithioate, **2**. Typically, a solution of **2** (39.9 mg, 0.1 mmol) and (-)-sparteine (4.7 µL, 0.02 mmol) in $CDCl_3$ (0.2 mL) was added to a solution of MAC (80.0 mg, 0.4 mmol) and **1** (14.8 mg, 0.04 mmol) in $CDCl_3$ (0.2 mL) before being stirred at room temperature for 1 h. Then, a solution of *L*-LA (0.46 g, 3.2 mmol), **1** (44.4 mg, 0.12 mmol) and (-)-sparteine (14.2 µL, 0.06 mmol) in $CDCl_3$ (4.2 mL) was added to the crude PMAC solution. The polymerization was allowed to stir at room temperature for 3 h. The product was purified by

precipitation in *n*-hexane three times and dried *in vacuo* to yield a pale yellow solid. ^{1}H NMR (400 Hz, $CDCl_3$, ppm, Figure S2) δ = 7.36 - 7.31 (4H, m, H5 & H6), 5.93 - 5.81 (4H, m, H12), 5.36 - 4.98 (2H PLLA + 2H PMAC + 2H CTA, m, H14 & H16, H13, H7), 4.63 - 4.60 (2H CTA + 2H PMAC, m, H4, H11), 4.40 - 4.22 (4H PMAC, m, H8 & H10), 3.36 (2H, t, $^{3}J_{H\text{-}H}$ = 7.5 Hz, H3), 1.75 - 1.41 (6H PLLA, H15 & H17), 1.35 - 1.19 (3H PMAC + 18H CTA, br, H9, H2); SEC ($CHCl_3$, PS standards): M_n = 5.8 kg·mol^{-1}, $Đ_M$ = 1.08.

Synthesis of PLLA-*b*-PMAC-*b*-PTHPA triblock copolymer, 4

Typically, THPA (2 g, 800 eq.) and PLLA-*b*-PMAC macro-initiator, **3** (93.1 mg, 1 eq.) were dissolved in $CHCl_3$ (2 mL) and transferred into a dried ampoule before adding AIBN (26.3 µL of a 10 mg/mL $CHCl_3$ solution). The solution was degassed by three freeze-pump-thaw cycles and sealed under argon and then placed in a 60 °C oil bath with stirring for 2.5 h. The product was purified by precipitation into *n*-hexane three times and dried *in vacuo* to give a pale yellow solid (31% conversion by ^{1}H NMR spectroscopy). ^{1}H NMR (400 MHz, $CDCl_3$, ppm, Figure 1) δ = 6.20 - 5.70 (1H PTHPA, br, H4), 5.35 - 5.00 (2H PLLA + 2H PMAC, m, H14 & H16, H13), 4.67 - 4.56 (2H PMAC, m, H12), 4.43 - 4.18 (4H PMAC, m, H9 & H11), 4.10 - 3.56 (2H PTHPA, br, H8), 2.73 - 2.23 (1H PTHPA, br, H2), 2.21 - 1.16 (8H PTHPA + 3H PMAC + 6H PLLA + 18 H dual-headed initiator, br, H3 & H5-H7, H10, H15 & H17, H1); SEC ($CHCl_3$, PS Standards): M_n = 46.1 kg·mol^{-1}, $Đ_M$ = 1.27.

Crystallization-driven self-assembly of PLLA-*b*-PMAC-*b*-PTHPA triblock copolymer, 4

The self-assembly of PLLA-*b*-PMAC-*b*-PTHPA triblock copolymer, **4**, was performed by a solvent evaporation method. Typically, 0.5 mL of THF and 2 mL of water (v_{THF} : v_{H2O} = 20 : 80) were added to 50 mg of polymer inside a vial. Acetic acid (1 eq. to each PTHPA block) was also added to the mixture to facilitate the hydrolysis of THPA. The vial was sealed with a needle inserted through the seal and the mixture was allowed to stir at 55 °C for 30 h before being quenched by cooling in liquid nitrogen and subsequent lyophilization. The freeze-dried nanoparticles were then dissolved directly into 18.2 MΩ·cm water (0.25 mg/mL) for TEM and DLS analysis.

Functionalization of PLLA-*b*-PMAC-*b*-PAA cylindrical micelles using photo-initiated thiol-ene radical addition

Generally, benzyl mercaptan and 2-benzyl-2-(dimethylamino)-4'-morpholinobutyrophenone UV initiator were directly added to an aqueous solution of PLLA-*b*-PMAC-*b*-PAA cylindrical micelles (4 mL, generally 3 mg/mL). THF or 1,4-dioxane (0.2 mL) was then added into the mixture (or without any further addition of organic solvent according to Table S1). The mixture was allowed to stir at room temperature for 1 h before exposure to UV irradiation for 1 h. The mixture was then dialyzed against 2% 1,4-dioxane in 18.2 MΩ·cm water overnight and then 18.2 MΩ·cm water for 2 days before subsequent lyophilization. The freeze-dried nanoparticles were directly dissolved in 18.2 MΩ·cm water for TEM analysis. For ^{1}H NMR spectroscopic analysis, the freeze-dried nanoparticles were dissolved in DMSO and precipitated in diethyl ether three times to completely remove unreacted thiols before being dried *in vacuo*. ^{1}H NMR (500 MHz, d_6-DMSO, ppm, Figure 4) δ = 13.6 -

11.2 (1H PAA, br, H4), 7.46 - 7.14 (4H dual-headed initiator + 5H benzyl mercaptan, m, H6 & H7, H19-H21), 5.93 - 5.79 (1H unfunctionalized PMAC, m, H13), 5.37 - 5.95 (2H PLLA, m, H22 & H24), 4.70 - 4.00 (6H PMAC, br, H9 & H11-H12, H15 & H17-H18), 2.35 - 2.12 (1H PAA, br, H2), 1.94 - 1.08 (2H PAA + 6H PLLA + 3H PMAC + 18H dual-headed initiator, br, H1, H3, H10 & H16, H23 & H25).

Functionalization of PLLA-*b*-PMAC-*b*-PAA triblock copolymers using photo-initiated thiol-ene radical reactions

Firstly, the precursor PLLA-*b*-PMAC-*b*-PTHPA triblock copolymer was added to a mixture of 1,4-dioxane and H_2O (2 mL and 0.5 mL respectively) with a further addition of acetic acid (3 *eq*. to each PTHPA block). The mixture was then sealed in a vial and heated at 65 °C to deprotect the PTHPA block. After 12 h, full deprotection was achieved as confirmed by ^{1}H NMR spectroscopy analysis. The mixture was precipitated in *n*-hexane three times and dried *in vacuo*. Benzyl mercaptan (10 *eq*. to each allyl group) and 2-benzyl-2-(dimethylamino)-4'-morpholinobutyrophenone UV initiator (0.5 *eq*. to each allyl group) were then added to PLLA-*b*-PMAC-*b*-PAA triblock copolymers (25 mg) in 1,4-dioxane (0.5 mL). The mixture was exposed to UV irradiation for 1 h before being precipitated in *n*-hexane three times and dried *in vacuo*. ^{1}H NMR (400 MHz, d_6-DMSO, ppm, Figure S7) δ = 12.7 - 11.7 (1H PAA, br, H4), 7.38 - 7.00 (4H dual-headed initiator + 5H benzyl mercaptan, m, H6 & H7, H13-H15), 5.27 - 4.95 (2H PLLA, m, H16 & H18), 4.60 - 3.95 (6H PMAC, br, H9 & H11-H12), 2.31 - 2.01 (1H PAA, br, H2), 1.86 - 1.02 (2H PAA + 6H PLLA + 3H PMAC + 18H dual-headed initiator, br, H1, H3, H10, H17 & H19).

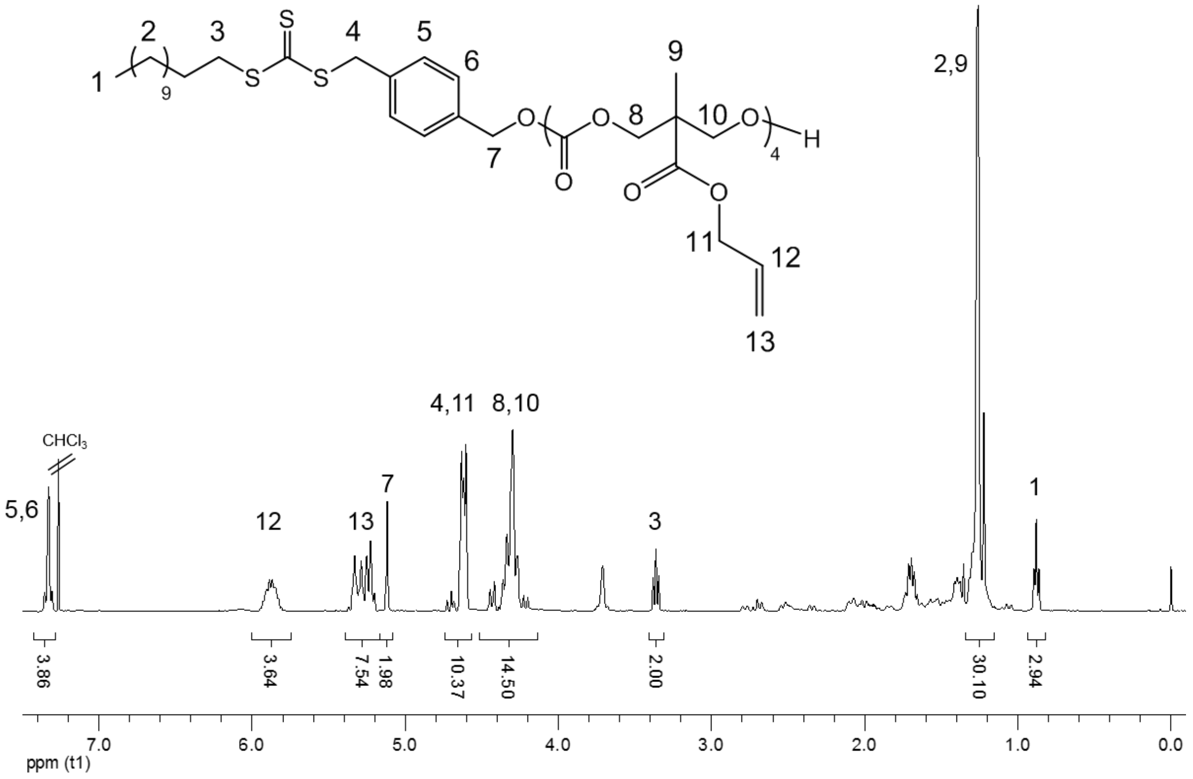

Figure S1. ^{1}H NMR spectrum (400 MHz, $CDCl_3$) of unpurified PMAC homopolymer.

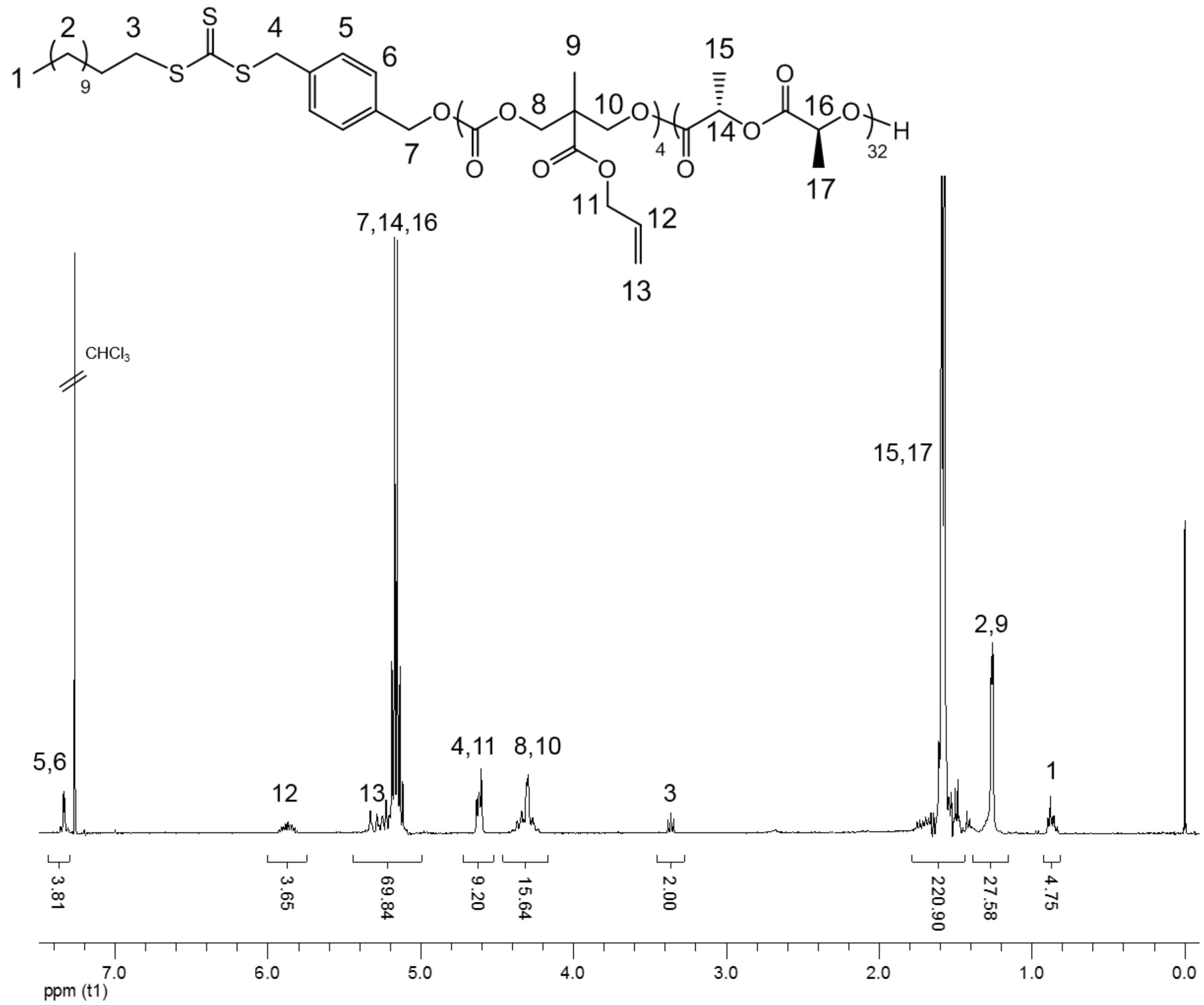

Figure S2. ^{1}H NMR spectrum (400 MHz, $CDCl_3$) of PLLA-*b*-PMAC diblock copolymer, **3**.

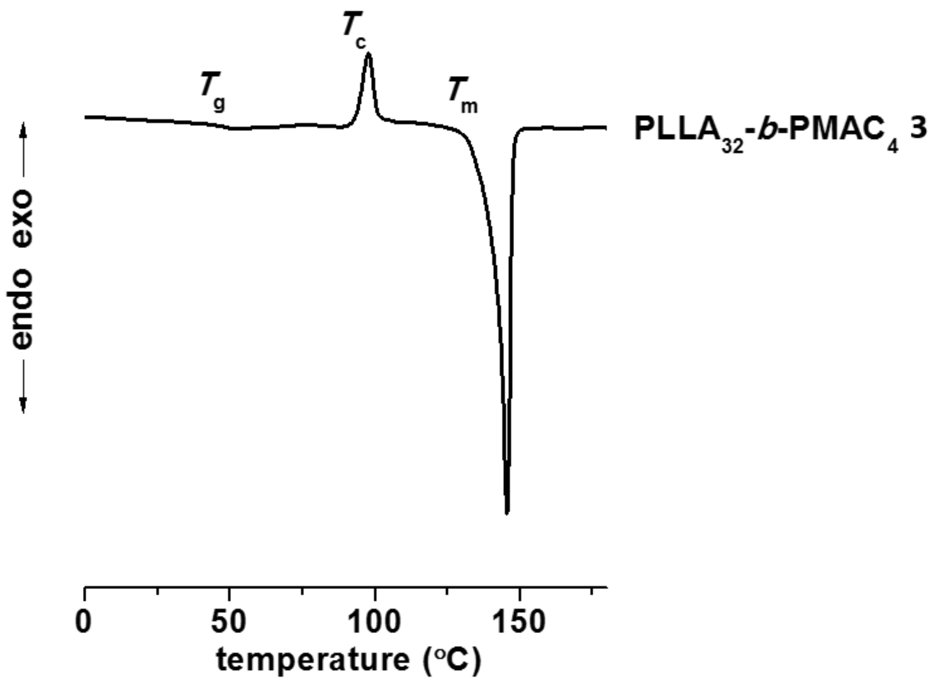

Figure S3. DSC thermogram (heating rate of 10 °C·min^{-1}) of PLLA-*b*-PMAC diblock copolymer, **3**.

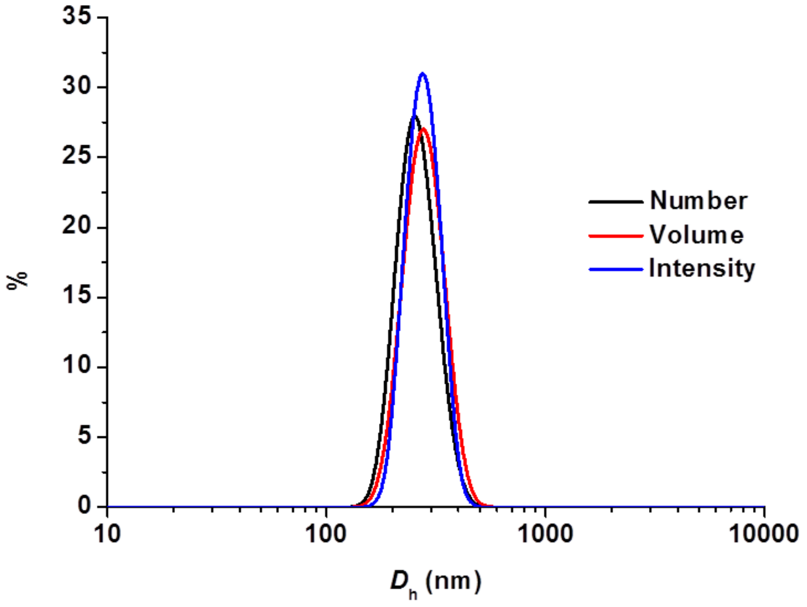

Figure S4. DLS data showing the distribution of PLLA-*b*-PMAC-*b*-PAA cylindrical micelles ($D_{h, av}$ = 268 nm, PD = 0.038).

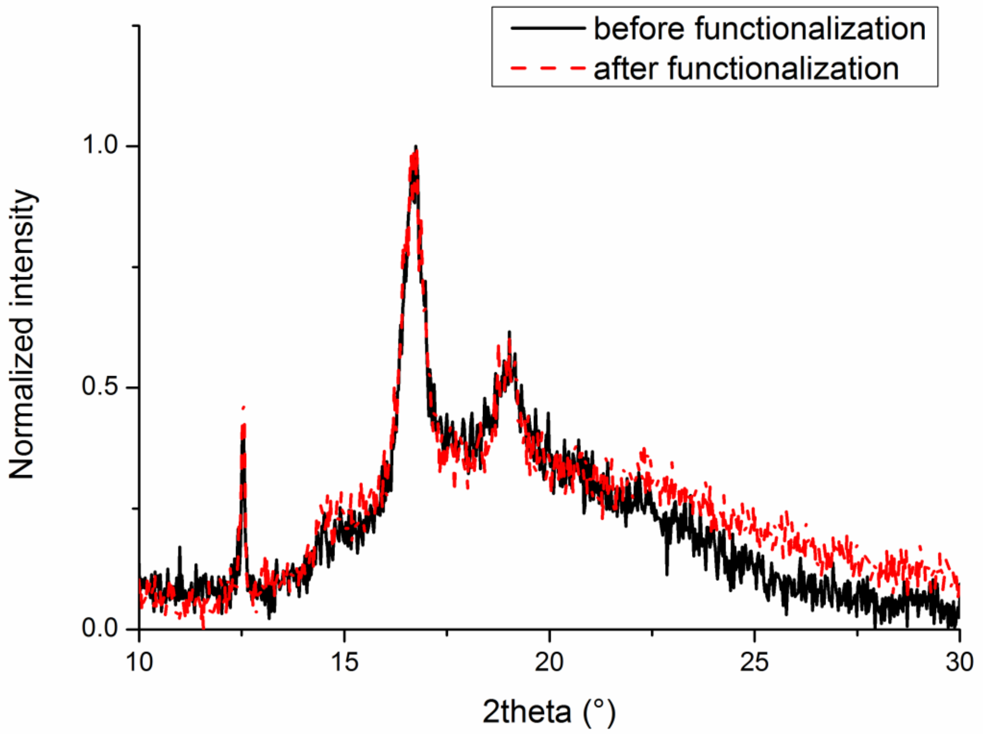

Figure S5. WAXD diffractogram showing the crystalline nature of the PLLA-*b*-PMAC-*b*-PAA cylindrical micelles and the same micelles after functionalization by thiol-ene chemistry.

Table S1. Functionalization of PLLA-*b*-PMAC-*b*-PAA cylindrical micelles with benzyl mercaptan or 6-(ferrocenyl)hexanethiol under different conditions using photo-initiated thiol-ene radical reactions

Cylindrical micelles concentration in H_2O (mg/mL)	Thiol	Additional solvent	Benzyl mercaptan (eq.)	UV initiator (eq.)	Functionalization ratio (%)[a]
3	benzyl mercaptan	—	10	2	74
3	benzyl mercaptan	THF	10	2	73
3	benzyl mercaptan	1,4-Dioxane	10	2	74
3	6-(ferrocenyl)hexanethiol	—	10	2	49

[a]Measured by ^{1}H NMR spectroscopy (400 MHz, d_6-DMSO).

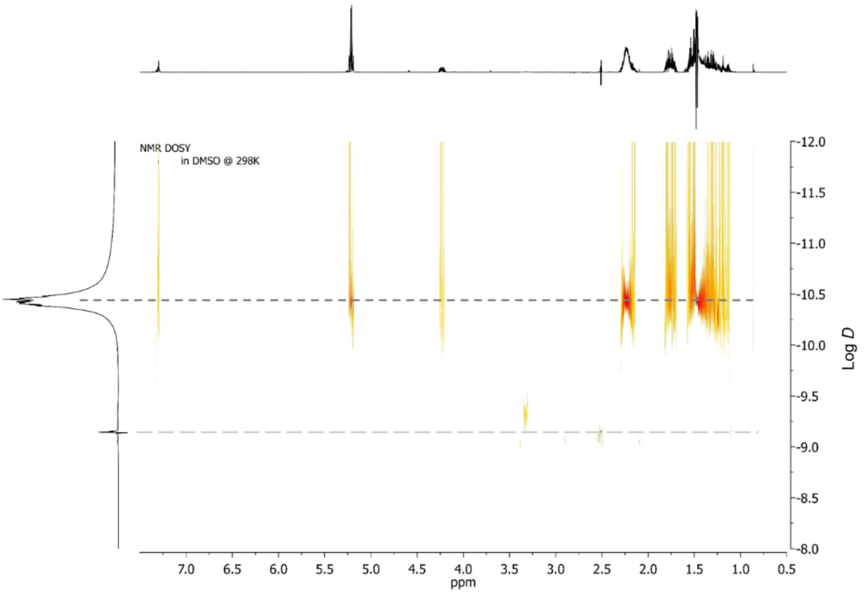

Figure S6. DOSY ^{1}H NMR spectrum (500 MHz, d_6-DMSO) of benzyl mercaptan functionalized PLLA-*b*-PMAC-*b*-PAA triblock unimers.

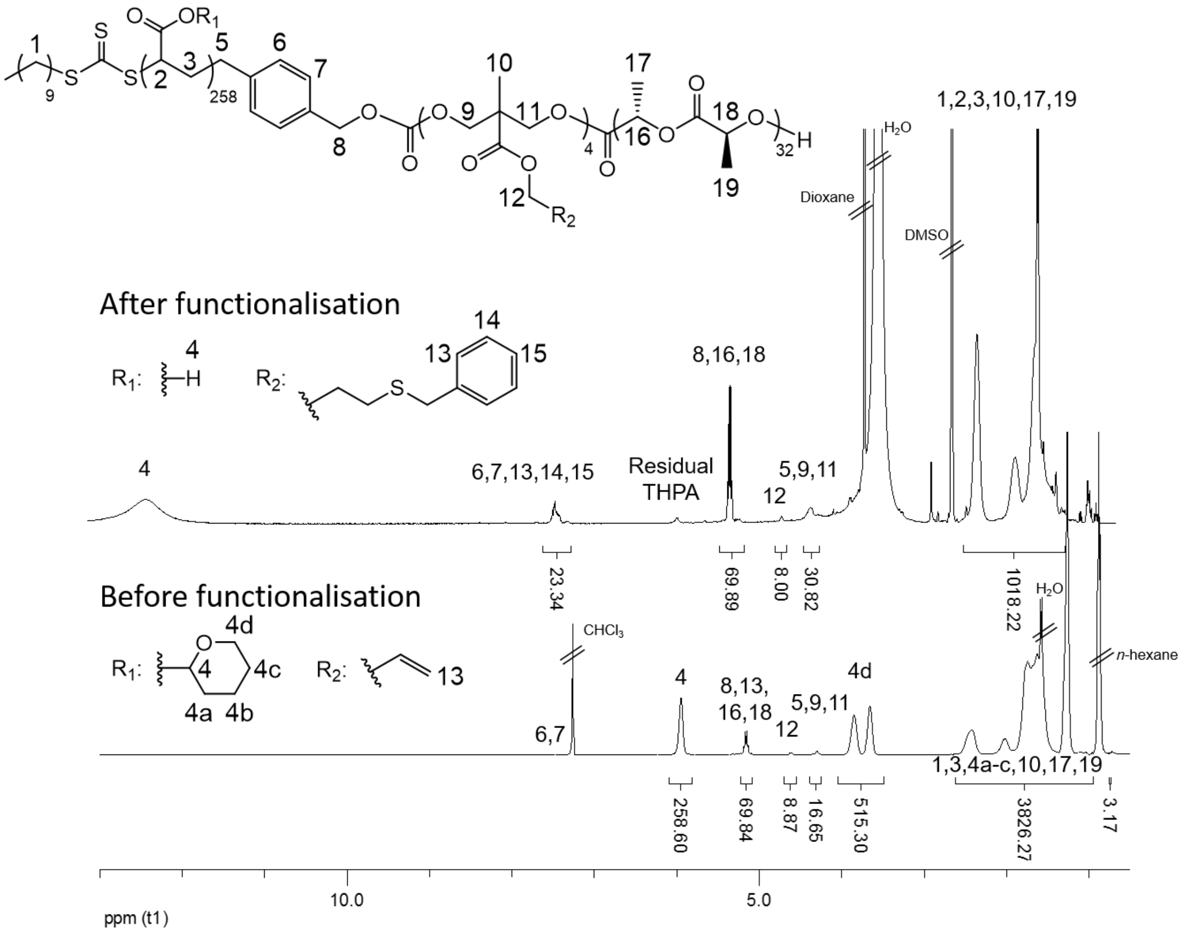

Figure S7. ^{1}H NMR spectrum (400 MHz) of benzyl mercaptan functionalized PLLA-*b*-PMAC-*b*-PAA triblock copolymer (top, d_6-DMSO) and non-functionalized PLLA-*b*-PMAC-*b*-PTHPA (bottom, $CDCl_3$).

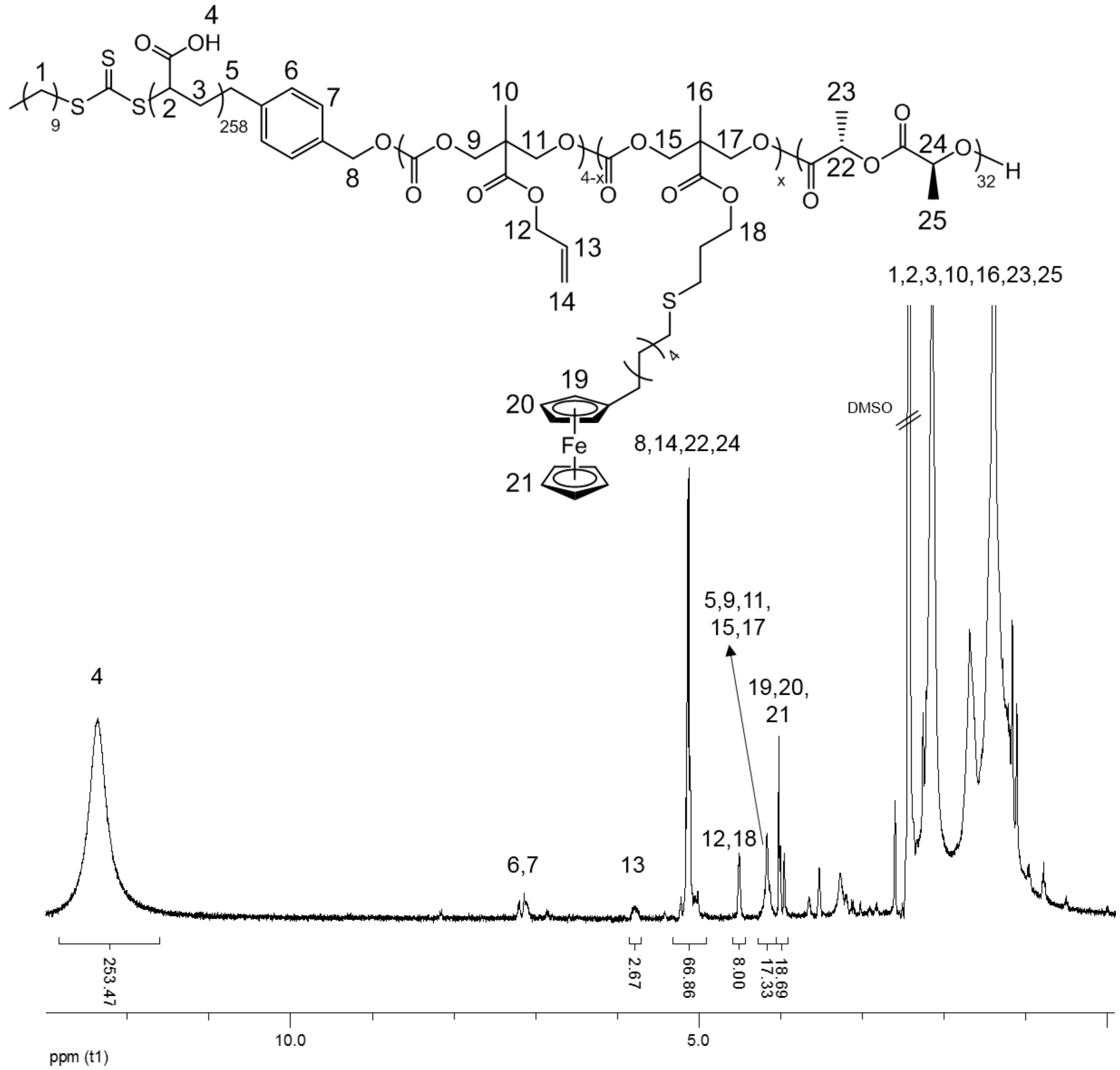

Figure S8. ^{1}H NMR spectrum (400 MHz, d_6-DMSO) of 6-(ferrocenyl)hexanethiol functionalized PLLA-*b*-PMAC-*b*-PAA triblock cylinders.

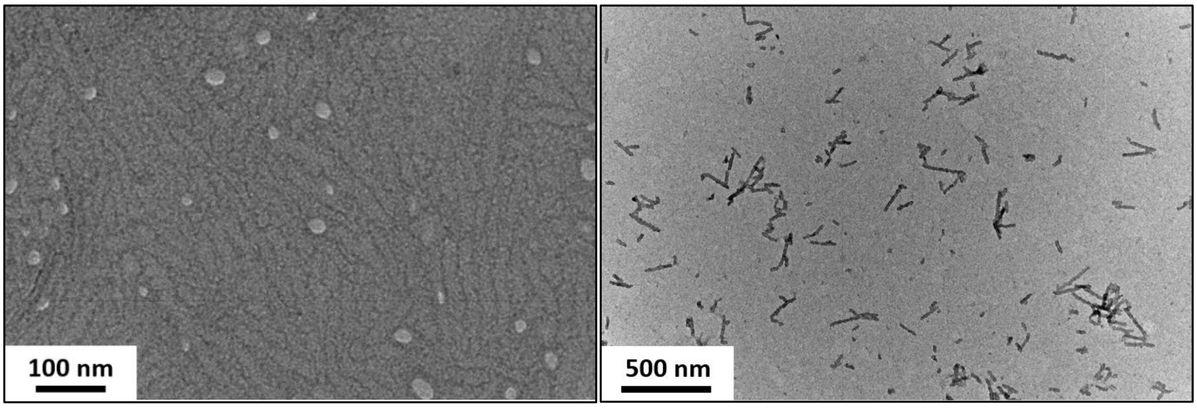

Figure S9. TEM image of PLLA-*b*-PMAC-*b*-PAA cylindrical micelles after functionalization with 6-(ferrocenyl)hexanethiol prepared: a. by freeze-drying 5 μL of the solution on a lacey carbon grid; b. by staining using UA on a Formvar grid.

References

1. R. C. Pratt, B. G. G. Lohmeijer, D. A. Long, P. N. P. Lundberg, A. P. Dove, H. Li, C. G. Wade, R. M. Waymouth and J. L. Hedrick, *Macromolecules*, 2006, **39**, 7863-7871.
2. *US Pat.,* 5 072 029, 1991.
3. R. K. O'Reilly, M. J. Joralemon, C. J. Hawker and K. L. Wooley, *Chem.--Eur. J.*, 2006, **12**, 6776-6786.
4. X. Hu, X. Chen, Z. Xie, S. Liu and X. Jing, *J. Polym. Sci., Part A: Polym. Chem.*, 2007, **45**, 5518-5528.
5. N. Petzetakis, A. P. Dove and R. K. O'Reilly, *Chem. Sci.*, 2011, **2**, 955-960.
